# Artificial intelligence–based 5‐year survival prediction and prognosis of DNp73 expression in rectal cancer patients

**DOI:** 10.1002/ctm2.159

**Published:** 2020-09-01

**Authors:** Tuan D. Pham, Chuanwen Fan, Hong Zhang, Xiao‐Feng Sun

**Affiliations:** ^1^ Center for Artificial Intelligence Prince Mohammad Bin Fahd University Khobar Saudi Arabia; ^2^ Department of Biomedical and Clinical Sciences Linköping University Linköping Sweden; ^3^ Department of Medical Sciences Örebro University Örebro Sweden

Dear Editor,

Preoperative radiotherapy (pRT) is known to improve local control for rectal cancer patients besides surgery.^1^, ^2^, ^3^ However, there are many patients who do not respond to pRT but experience side effects. It is therefore urgently required to find promising pRT‐related biomarkers for approaching precision medicine.

In this study, we investigated the application of artificial intelligence (AI) for discovering the predictive and prognostic power of the DNp73 expression in a cohort of 143 rectal cancer patients from the Swedish rectal cancer trial of pRT.^2^ The DNp73 expression was identified by immunohistochemistry (IHC), and the procedure for the IHC image extraction was described in Ref. 4. While the manual pathology‐based analysis of DNp73 expression did not provide any survival information (P>.05), the average AI‐based validation results show very high accuracy rates (≥93%) for the 5‐year prediction and prognosis of the rectal cancer patients either with or without pRT.

The DNp73 expression was investigated in 96 biopsies, surgically resected normal and tumor samples from 77 patients without pRT and 59 patients with pRT (Figure 1A,B). The DNp73 staining was performed in the whole group of surgically resected distant normal (n=119), adjacent normal (n=79), and tumor samples (n=136). Strong cytoplasmic DNp73 staining was present in the normal and tumor cells (Figure 1A,B). In the analysis of the clinicopathologic and biologic significance of DNp73 expression, we divided the patients into DNp73 weak and strong groups. The expression of DNp73 was significantly increased in the tumors either without or with pRT, when compared with the normal mucosa (Figure 1C, P<.001). The significant differences of the DNp73 expression were observed in the matched cases of the distant normal mucosa, adjacent normal mucosa, and tumor derived from the same patient (Figure 1D, P=.002). We found that the DNp73 expression in the biopsies was not related to any clinicopathologic variables including gender, age, differentiation, surgical type, local recurrence, distant recurrence, and survival status (Table S1 in the Supporting Information, P>.05), while the DNp73 expression was related to local recurrence (Table S2 in the Supporting Information, P=.042) in the surgically resected tumor samples with pRT and surgical type (Table S2, P=.021) in the surgically resected tumor samples without pRT.

**FIGURE 1:**
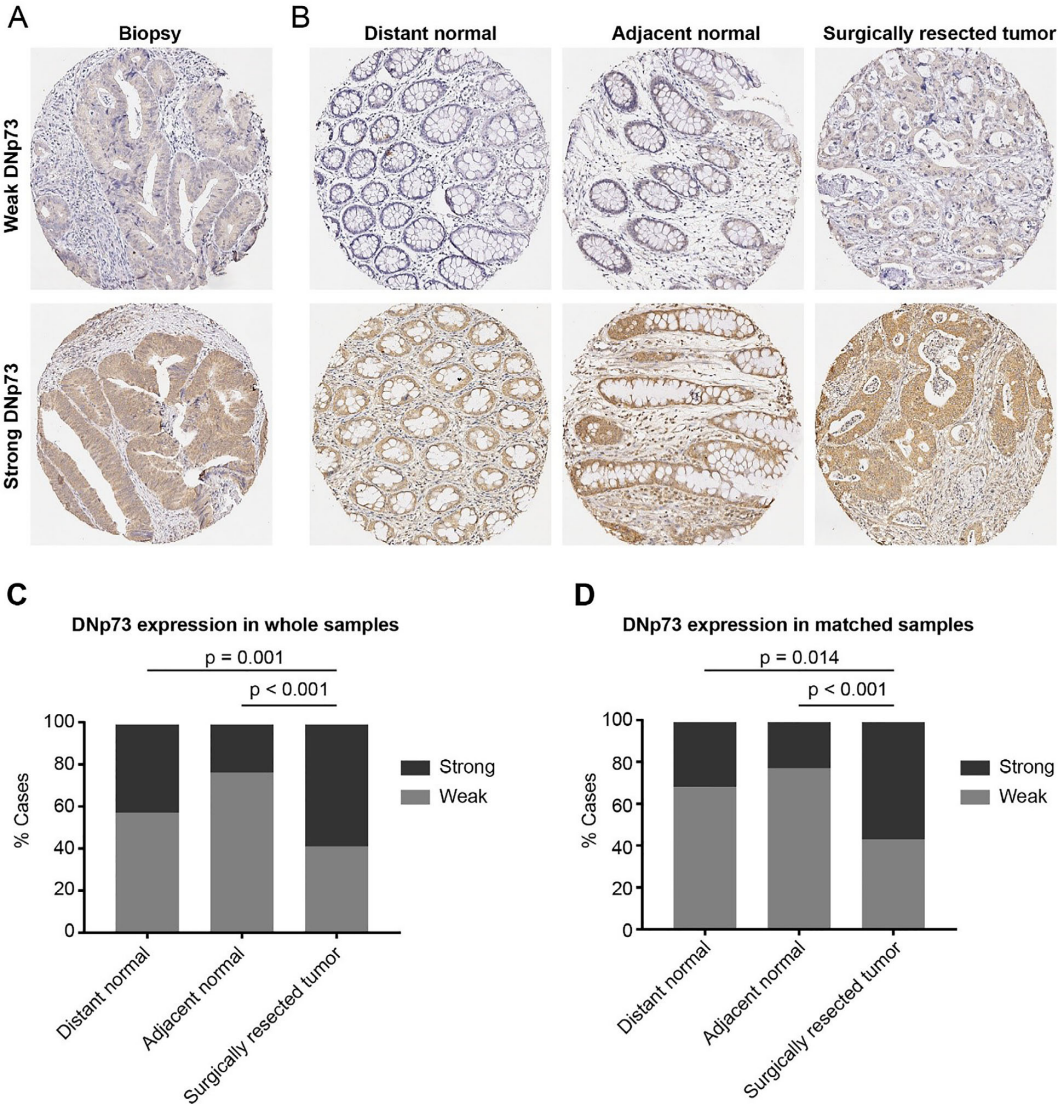
DNp73 expression by IHC staining in tumor samples from rectal cancer patients. A representative IHC image of DNp73 expression in biopsies (A) and surgically resected samples, including distant normal mucosa, adjacent normal mucosa, and surgically resected tumor (B); DNp73 expression in distant normal mucosa, adjacent normal mucosa, and surgical tumor obtained from whole samples (C), and matched samples (D). Whole samples indicated all surgically resected samples. Matched samples included surgically resected samples (including distant normal, adjacent normal, and primary tumor samples) from the same patient

Because AI is considered as the foremost advanced approach in cancer research,^5^, ^6^, ^7^, ^8^, ^9^, ^10^ we then used AI methods for exploring the DNp73 expression with respect to 5‐year survival prediction and prognosis. The methods consist of 10 pretrained convolutional neural networks (CNNs) whose properties are listed in Table S3 in the Supporting Information. The data processing and network configuration are described as follows. Each whole IHC image was resized to match the input image size specified by each of the 10 networks (see the last column of Table S3). In performing the transfer learning, parameters of the networks were set as stochastic gradient descent with momentum = 0.9, minimum batch size = 10, maximum number of epochs = 6, initial learning rate = 0.0003, data were shuffled before every training epoch, learning rate drop factor = 0.1, learning rate drop period = 10, factor for the $L_{2}$ regularizer = 0.0001, and the method used for gradient thresholding = $L_{2}$ norm. The training and testing of the datasets for biopsies and surgically resected tumors without or with pRT were carried out by randomly selecting 90% of each dataset for training the CNN models and the remaining 10% for validation. Both training and validation of the 10 CNNs were repeated 10 times.

Average results and standard deviations for the accuracy, >5‐year (defined as true positive rate), and ≤5‐year (defined as true negative rate) prediction and prognosis (see Table S4 for definitions in the Supporting Information) obtained from selected top‐performance CNNs, whose average accuracy ≥93%, are shown in Table 1. As a case for using DenseNet201 for biopsies without pRT, the average prediction for >5 years = 90%, ≤5 years = 98%, with average accuracy = 96%; and for biopsies with pRT, the average prediction for >5 years = 97%, ≤5 years = 80%, with average accuracy = 93%. Using ResNet101 for surgically resected tumors without pRT, the average prediction for >5 years = 98%, ≤5 years = 90%, with average accuracy = 96%. Using DenseNet201 for the tumors with pRT, the average prediction for >5 years = 93%, ≤5 years = 95%, with average accuracy = 93%.

**TABLE 1:** AI‐based prediction and prognosis of DNp73 expression

CNN model	Accuracy (%)	>Five years (%)	≤Five years (%)
	Biopsies without preoperative radiotherapy		
ResNet50	94.00 ± 9.66	70.00 ± 48.30	100.00 ± 0.00
VGG16	94.00 ± 9.66	80.00 ± 42.16	97.50 ± 7.91
DenseNet201	96.00 ± 8.43	90.00 ± 31.62	97.50 ± 7.91
	Biopsies with preoperative radiotherapy		
ResNet101	92.50 ± 12.08	100.00 ± 0.00	70.00 ± 48.30
DenseNet201	92.50 ± 16.87	96.67 ± 10.54	80.00 ± 42.16
	Tumors without preoperative radiotherapy		
GoogleNet	94.29 ± 18.07	96.00 ± 12.65	90.00 ± 31.63
ResNet50	94.29 ± 12.05	96.00 ± 8.43	90.00 ± 21.08
DenseNet201	94.29 ± 13.80	96.00 ± 8.43	90.00 ± 31.62
InceptionV3	94.29 ± 12.05	100.00 ± 0.00	80.00 ± 42.16
ResNet101	95.71 ± 9.64	98.00 ± 6.32	90.00 ± 21.08
	Tumors with preoperative radiotherapy		
ResNet101	90.00 ± 16.10	92.50 ± 12.08	85.00 ± 33.74
InceptionV3	93.33 ± 11.65	95.00 ± 10.54	90.00 ± 31.62
DenseNet201	93.33 ± 16.10	92.50 ± 16.87	95.00 ± 15.81
NasNetLarge	93.33 ± 16.10	95.00 ± 15.81	90.00 ± 21.08

The results obtained from other CNNs for the prediction and prognosis using the biopsies and tumors are shown in Table S1. Figures S1 and S2 (in the Supporting Information) show a training process and features learned by DenseNet‐201 for classifying the biopsies without pRT, respectively. Using the maximum number of epochs = 6 for training, the accuracy could reach 100% (Figure S1).

These present results have a useful implication that DNp73 expression, by examining either biopsies or surgical tumors, can determine the prediction or prognosis of the patients without pRT or with pRT. More interestingly, for the first time, we report an accurate AI‐based classification of the biopsy IHC‐staining images and its correlation of 5‐year prognosis, which is expected to be of benefit for clinical treatment decision, rather than traditional IHC assay.

## AUTHOR CONTRIBUTIONS

TDP, CWF, HZ, and XFS designed the research; TDP conceptualized and performed the study of AI; CWF, HZ, and XFS provided the data; TDP, CWF, HZ, and XFS contributed to the analysis of the results; and TDP, CWF, and XFS wrote the manuscript.

## CONFLICT OF INTEREST

The authors declare no competing interest.

## Supporting information

Supporting Information

## Data Availability

The IHC data and Matlab code used in this study are deposited at https://sites.google.com/view/tuan-d-pham/codes.
