# Supplementary material for: Artificial intelligence–based 5‐year survival prediction and prognosis of DNp73 expression in rectal cancer patients
Source: Clin Transl Med. 2020 Sep 1;10(4):e159. doi: 10.1002/ctm2.159 (PMC7507021; doi:10.1002/ctm2.159)
Supplement: Supplementary file 1 — Supporting Information [file CTM2-10-e159-s001.pdf]

Table S1: Correlation of DNp73 expression and clinical characteristics in biopsies of rectal cancer patients.

Characteristics	Weak (%)	Strong (%)	p-value
Gender			0.097
Male	26 (46)	30 (54)	
Female	26 (63)	15 (37)	
Age			0.211
≤ 67	20 (47)	23 (53)	
> 67	32 (59)	22 (41)	
TNM stage			0.059
I	17 (55)	14 (45)	
II	18 (67)	9 (33)	
III	11 (35)	20 (65)	
IV	6 (75)	2 (25)	
Differentiation			0.221
Good	3 (60)	2 (40)	
Moderate	40 (52)	37 (48)	
Poor	3 (50)	3 (50)	
Unknown	6 (67)	3 (33)	
Surgical type			0.743
Rectal amputation	26 (55)	21 (45)	
Abdominoperineal	26 (52)	24 (48)	
Local recurrence			0.250
Yes	6 (40)	9 (60)	
No	46 (56)	36 (44)	
Distant metastasis			0.333
Yes	17 (47)	19 (53)	
No	35 (57)	26 (43)	
Dead			0.467
Yes	16 (48)	17 (52)	
No	36 (56)	28 (44)	

Table S2: Correlation of DNp73 expression in surgically resected tumors and clinical characteristics in rectal cancer patients without preoperative radiotherapy (pRT) or with pRT.

	DNp73 expression in patients					
	with pRT (%)			without pRT (%)		
Characteristics	Weak	Strong	*p*-value	Weak	Strong	*p*-value
Gender			0.871			0.233
Male	18 (50)	18 (50)		21 (49)	22 (51)	
Female	11 (48)	12 (52)		12 (35)	22 (65)	
Age			0.920			0.690
$\leq$67	11 (38)	11 (38)		15 (45)	18 (55)	
>67	18 (44)	19 (46)		18 (41)	26 (59)	
TNM stage			0.999			0.217
I	8 (50)	8 (50)		7 (33)	14 (67)	
II	10 (48)	11 (52)		8 (42)	11 (58)	
III	9 (50)	9 (50)		18 (53)	16 (47)	
IV	2 (50)	2 (50)		0 (0)	3 (100)	
Differentiation			0.213			0.272
Good	0 (0)	3 (100)		1 (20)	4 (80)	
Moderate	20 (53)	18 (47)		27 (48)	29 (52)	
Poor	9 (50)	9 (50)		5 (31)	11 (69)	
Surgical type			0.472			0.021
Rectal amputation	9 (43)	12 (57)		23 (55)	19 (45)	
Abdominoperineal	20 (53)	18 (47)		10 (29)	25 (71)	
Local recurrence			0.042			0.692
Yes	0 (0)	4 (100)		8 (47)	9 (53)	
No	29 (53)	26 (47)		25 (42)	35 (58)	
Distant metastasis			0.341			0.893
Yes	10 (42)	14 (58)		13 (42)	18 (58)	
No	19 (54)	16 (46)		20 (43)	26 (57)	
Dead			0.207			0.946
Yes	8 (38)	13 (62)		13 (43)	17 (57)	
No	21 (55)	17 (45)		20 (43)	27 (57)	

Table S3: Some properties of ten pre-trained CNNs. Note: *NasNetLarge network does not consist of a linear sequence of modules.

CNN	Depth	Size (MB)	Parameters (millions)	Input image size
AlexNet	8	227	61.0	227 × 227
GoogleNet	22	27	7.0	224 × 224
SqueezeNet	18	4.6	1.24	227 × 227
ResNet50	50	96	25.6	224 × 224
ResNet101	101	167	44.6	224 × 224
InceptionV3	48	89	23.9	299 × 299
InceptionResNetV2	164	209	55.9	299 × 299
VGG16	16	515	138	224 × 224
DenseNet201	201	77	20.0	224 × 224
NasNetLarge	*	360	88.9	331 × 331

Table S4: Statistical measures for the prediction and prognosis of DNp73 expression.

Terminology	Definition
True positive (TP)	Number of patients correctly identified as having survival rate > 5 years.
True negative (TN)	Number of patients correctly identified as having survival rate ≤ 5 years.
P	Total number of patients whose survival rates > 5 years.
N	Total number of patients whose survival rates ≤ 5 years.
True positive rate (TPR)	$TPR = TP/P$.
True negative rate (TNR)	$TNR = TN/N$.
Accuracy (ACC)	$ACC = (TP + TN)/(P + N)$.

Table S5: Other AI-based prediction and prognosis of DNp73 expression.

CNN model	Accuracy(%)	> Five years (%)	≤ Five years (%)
	Biopsies without preoperative radiotherapy		
InceptionResNetV2	80.00 ± 0.00	0.00 ± 0.00	100.00 ± 0.00
SqueezeNet	82.00 ± 11.35	20.00 ± 42.16	97.50 ± 7.91
GoogleNet	84.00 ± 20.66	70.00 ± 48.30	87.50 ± 21.25
AlexNet	88.00 ± 25.30	70.00 ± 48.30	92.50 ± 23.72
InceptionV3	88.00 ± 10.33	60.00 ± 51.64	95.00 ± 10.54
ResNet101	92.00 ± 13.98	70.00 ± 48.30	97.50 ± 7.91
NasNetLarge	92.00 ± 10.33	60.00 ± 51.64	100.00 ± 0.00
	Biopsies with preoperative radiotherapy		
VGG16	62.50 ± 41.25	66.67 ± 47.14	50.00 ± 52.70
SqueezeNet	70.00 ± 19.72	90.00 ± 22.50	10.00 ± 31.62
InceptionResNetV2	72.50 ± 7.91	96.67 ± 10.54	0.00 ± 0.00
ResNet50	82.50 ± 12.08	96.67 ± 10.54	40.00 ± 51.64
GoogleNet	85.00 ± 17.48	96.67 ± 10.54	50.00 ± 52.70
InceptionV3	85.00 ± 12.91	100.00 ± 0.00	40.00 ± 51.64
NasNetLarge	85.00 ± 12.91	100.00 ± 0.00	40.00 ± 51.64
AlexNet	87.50 ± 13.18	96.67 ± 10.54	60.00 ± 51.64
	Tumors without preoperative radiotherapy		
VGG16	71.43 ± 25.20	72.00 ± 37.95	70.00 ± 42.16
AlexNet	81.43 ± 26.98	82.00 ± 30.48	80.00 ± 42.17
SqueezeNet	81.43 ± 15.13	88.00 ± 21.50	65.00 ± 41.16
NasNetLarge	90.00 ± 21.35	92.00 ± 19.32	85.00 ± 33.75
InceptionResNetV2	91.42 ± 13.80	100.00 ± 0.00	70.00 ± 48.30
	Tumors with preoperative radiotherapy		
SqueezeNet	70.00 ± 15.32	90.00 ± 12.91	30.00 ± 34.96
VGG16	81.67 ± 22.84	87.50 ± 31.73	70.00 ± 42.16
AlexNet	83.33 ± 23.57	87.50 ± 17.68	75.00 ± 42.49
InceptionResNetV2	86.67 ± 21.94	90.00 ± 17.48	80.00 ± 34.96
GoogleNet	88.33 ± 17.66	92.50 ± 12.08	80.00 ± 42.16
ResNet50	88.33 ± 15.81	92.50 ± 16.87	80.00 ± 34.96

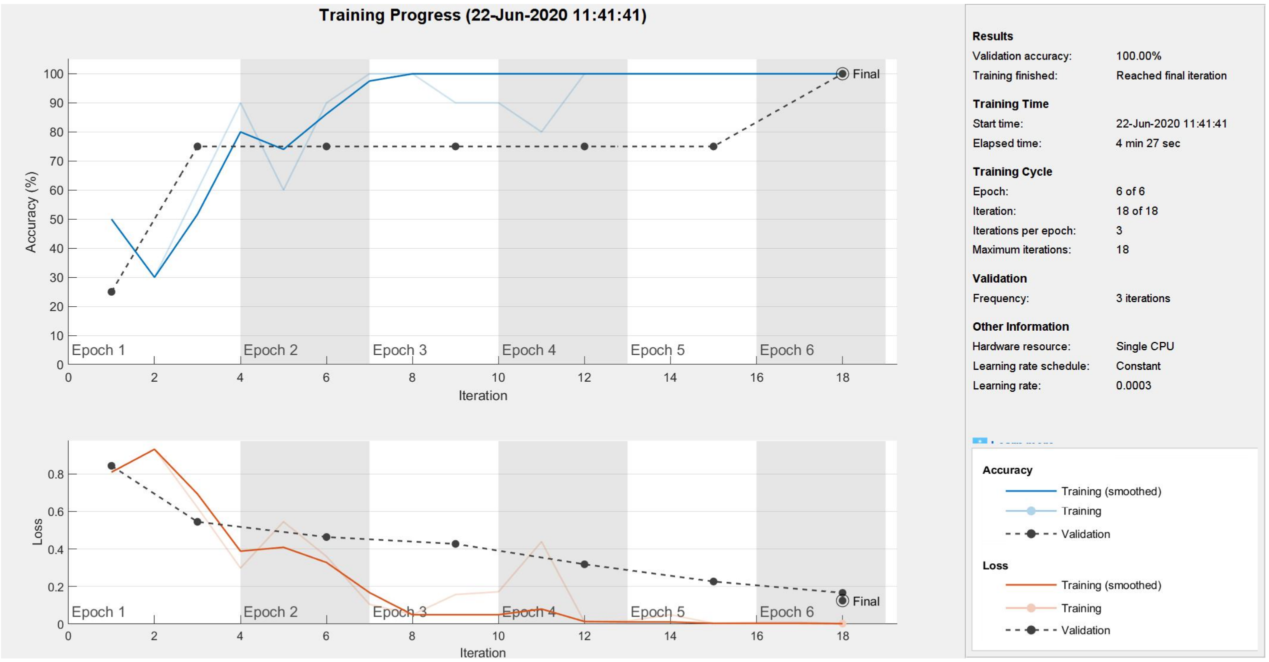

Figure S1: A training process of DenseNet-201 for classification of biopsies without preoperative radiotherapy.

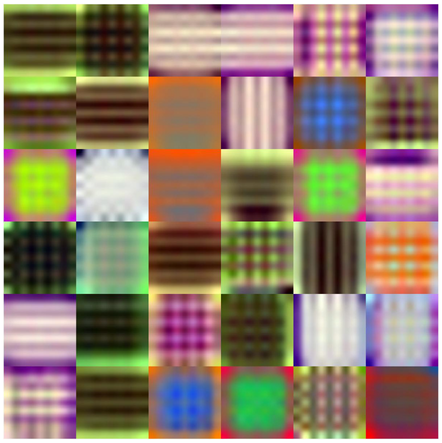

(a) Layer ’conv1—conv’(convolution)

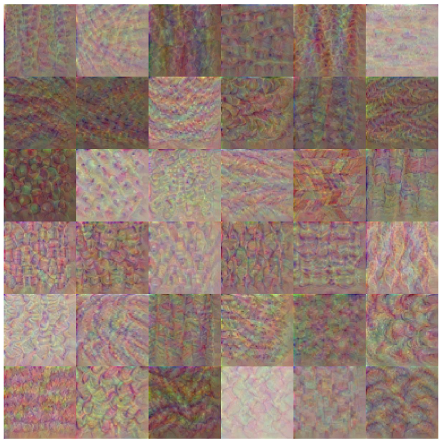

(b) Layer ‘conv4_block7_1_conv’ (convolution)

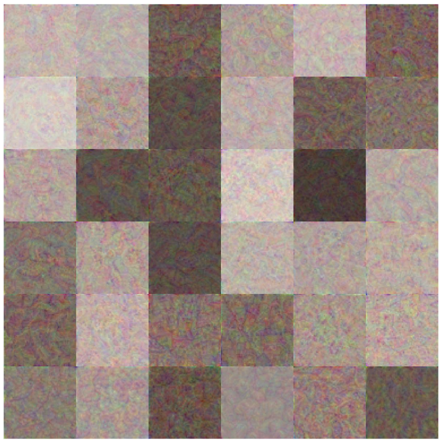

(c) Layer ‘conv5_block9_1_conv’ (convolution)

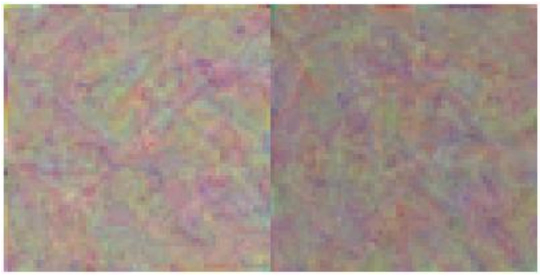

(d) Layer ‘new_fc’ (fully connected)

Figure S2: Features learned by DenseNet-201 for classification of biopsies without preoperative radiotherapy: 36 features in (a), (b), and (c), and 2 features in (d).
